# Structural and Functional Characterization of the Type Three Secretion System (T3SS) Needle of *Pseudomonas aeruginosa*

**DOI:** 10.3389/fmicb.2019.00573

**Published:** 2019-03-29

**Authors:** Charlotte Lombardi, James Tolchard, Stephanie Bouillot, Luca Signor, Caroline Gebus, David Liebl, Daphna Fenel, Jean-Marie Teulon, Juliane Brock, Birgit Habenstein, Jean-Luc Pellequer, Eric Faudry, Antoine Loquet, Ina Attrée, Andréa Dessen, Viviana Job

**Affiliations:** ^1^Univ. Grenoble Alpes, CEA, CNRS, Institut de Biologie Structurale (IBS), Grenoble, France; ^2^Institute of Chemistry and Biology of Membranes and Nanoobjects, Institut Européen de Chimie et Biologie (CBMN), UMR5248 CNRS, University of Bordeaux, Pessac, France; ^3^Univ. Grenoble Alpes, Bacterial Pathogenesis and Cellular Responses Group, U1036 INSERM, ERL5261 CNRS, CEA, Grenoble, France; ^4^Brazilian Biosciences National Laboratory (LNBio), Centro Nacional de Pesquisa em Energia e Materiais (CNPEM), Campinas, Brazil

**Keywords:** type III secretion system, *Pseudomonas aeruginosa*, T3SS needle, structure, virulence, immunofluorescence microscopy, mutagenesis

## Abstract

The type three secretion system (T3SS) is a macromolecular protein nano-syringe used by different bacterial pathogens to inject effectors into host cells. The extracellular part of the syringe is a needle-like filament formed by the polymerization of a 9-kDa protein whose structure and proper localization on the bacterial surface are key determinants for efficient toxin injection. Here, we combined *in vivo*, *in vitro*, and *in silico* approaches to characterize the *Pseudomonas aeruginosa* T3SS needle and its major component PscF. Using a combination of mutagenesis, phenotypic analyses, immunofluorescence, proteolysis, mass spectrometry, atomic force microscopy, electron microscopy, and molecular modeling, we propose a model of the *P. aeruginosa* needle that exposes the N-terminal region of each PscF monomer toward the outside of the filament, while the core of the fiber is formed by the C-terminal helix. Among mutations introduced into the needle protein PscF, D76A, and P47A/Q54A caused a defect in the assembly of the needle on the bacterial surface, although the double mutant was still cytotoxic on macrophages in a T3SS-dependent manner and formed filamentous structures in *vitro*. These results suggest that the T3SS needle of *P. aeruginosa* displays an architecture that is similar to that of other bacterial needles studied to date and highlight the fact that small, targeted perturbations in needle assembly can inhibit T3SS function. Therefore, the T3SS needle represents an excellent drug target for small molecules acting as virulence blockers that could disrupt pathogenesis of a broad range of bacteria.

## Introduction

Bacterial pathogens have developed different strategies to colonize, invade and kill eukaryotic cells. One of the most successful mechanisms involves the use of the type III secretion system (T3SS), a macromolecular complex present on the surface of numerous pathogenic and colonizing species such as *Salmonella*, *Shigella*, *enteropathogenic Escherichia coli* (EPEC), *Yersinia*, and *Pseudomonas* spp. (Galán and Collmer, 1999; Galán et al., 2014; Wagner et al., 2018). The T3SS can be described as a multicomponent protein structure consisting of four major constituents: (i) a basal body that anchors the system to the bacterial membranes; (ii) an export apparatus which includes a cytoplasmic sorting platform that selects substrates and provides energy for the secretion process; (iii) a needle filament which protrudes toward the outside of the bacterial surface and serves as a passage for translocator proteins and effectors; and (iv) a translocation pore formed in the eukaryotic cell membrane that allows the entry of virulence effectors into the host cytoplasm.

The basal body and the needle form the so-called needle complex (NC), which represents the core structure of the T3SS. The number of NCs per bacterial cell varies between species. For instance, *Salmonella typhimurium*, assembles between 10 and 100 NCs per cell (Kubori et al., 1998), while only a few NCs were detected on the surface of *Pseudomonas aeruginosa* (Perdu et al., 2015). Notably, the T3SS needle is formed by a single polymerized protein that has been studied in the context of vaccine development against several pathogens (Charro and Mota, 2015; Jneid et al., 2016; Koroleva et al., 2017). It has been shown that immunization with needle proteins induces specific humoral and T cell responses and abrogates bacterial pathogenicity in animal models (Koroleva et al., 2017). In addition, small molecules such as phenoxyacetamide, that are directed against specific residues of the needle protein PscF can inhibit *P. aeruginosa* T3SS secretion (Bowlin et al., 2014) and the same compound also inhibits abscess formation in mice by directly blocking the T3SS (Berube et al., 2017). These results highlight the need for further characterization of molecular aspects and structures of NC assemblies to develop more specific anti-microbial molecules (Izoré et al., 2011).

Formation of the T3SS needle is a spatio-temporally regulated process. Protein monomers of the T3SS needle from different bacterial species have an approximate molecular weight of 9-kDa and display 13% sequence identity and 42% sequence similarity (considering the main human pathogens: *Salmonella, Shigella, P. aeruginosa*, and *Yersinia* sp.) (Kubori et al., 2000; Hoiczyk and Blobel, 2001; Pastor et al., 2005). Prior to secretion and assembly on the bacterial surface the spontaneous assembly of needle monomers in the bacteria cytosol is prevented by binding to two distinct chaperones concomitantly (Quinaud et al., 2005; Sun et al., 2008; Sal-Man et al., 2013), as is also the case for translocator proteins (Job et al., 2010; Discola et al., 2014; Nguyen et al., 2015). High resolution structures of T3SS needle proteins from different bacteria that have been solved to date (Deane et al., 2006; Quinaud et al., 2007; Wang et al., 2007; Sun et al., 2008; Poyraz et al., 2010) by crystallography and solution NMR reveal a similar protein fold composed of a helix-turn-helix motif with two long helices. Furthermore, one notable feature of T3SS needle proteins is an amphipathic C-terminal helix, which is ‘trapped’ within a hydrophobic concave region of the dual chaperone interface, presumably in order to block self-association (and early polymerization) (Quinaud et al., 2007; Sun et al., 2008). Upon T3SS activation, the needle protein dissociates from its chaperones, binding to the inner rod protein within the basal body (Kuhlen et al., 2018). Polymerization then occurs through addition of 100s of monomers to the distal end of growing needle. Interestingly, some bacteria possess accessory proteins that aid in assembly of the needle filament and accelerate the polymerization process *in vitro*, such as recently reported for *Salmonella typhimurium* (Kato et al., 2018). The final assembled needle in all bacteria is then capped by a tip protein (or soluble translocator), which is absolutely necessary to allow the formation of the translocation pore for the injection of effectors (Matteï et al., 2011; Harmon et al., 2013). During cell contact the specific needle length is essential for proper function yet the exact mechanism of length control still remains controversial (Cornelis, 2006). It has been suggested that a molecular ruler is involved (Kubori et al., 2000; Journet et al., 2003; Wagner et al., 2010; Bergeron et al., 2016), or that needle length is regulated by a precise timing of substrate switching, as suggested by recent mathematical models (Nariya et al., 2016).

The length of a fully assembled T3SS needle varies between bacterial species from 45 to 80 nm (Kubori et al., 1998; Tamano et al., 2000; Pastor et al., 2005; Radics et al., 2014; Park et al., 2018) while the outer and inner diameter range from 5–13 and 2–2.5 nm, respectively (Kubori et al., 2000; Pastor et al., 2005; Poyraz et al., 2010; Fujii et al., 2012; Loquet et al., 2012; Demers et al., 2013; Radics et al., 2014; Hu et al., 2018). Solid-state NMR structures of the T3SS needles of PrgI from *Salmonella* (Loquet et al., 2012) and MxiH from *Shigella* (Demers et al., 2014) indicated a super helical multimeric structure with 5.7 subunits per turn (Loquet et al., 2012) composed of three intermolecular subunit–subunit interfaces, one of which is axial (subunit i to i+11) and two are lateral (subunit i to i+5/6). Interestingly, the orientation of the two aforementioned helices within the needle structure has been the subject of debate (Fujii et al., 2012; Demers et al., 2013, 2014; Verasdonck et al., 2015). The first cryo-electron microscopy studies performed on the *Shigella* T3SS suggested that the C-terminus of each protomer points toward the outside (Deane et al., 2006; Fujii et al., 2012), while studies involving a combination of cryo-EM and solid state NMR indicated that at least in *Salmonella* and *Shigella* T3SS needles have the N-terminus that points toward the outside of the super-helical structure (Demers et al., 2013, 2014; Verasdonck et al., 2015).

In this work we address the architecture of the T3SS needle of *P. aeruginosa* using a combination of mutagenesis followed by phenotypic studies, proteolysis, mass spectrometry (MS), electron and atomic force microscopies (EM and AFM) and molecular modeling. We show that the *P. aeruginosa* T3SS needle monomer PscF has an N-terminal region oriented toward the outside of the filament, while the C-terminal helix of PscF forms the needle core. Using mutagenesis, we found that D76 is essential for needle polymerization as well as its assembly on the bacterial surface, while residues P47 and Q54 are concomitantly dispensable for polymerization but affect needle stability and effector secretion. Our results, analyzed in the context of other published studies, suggest that a common architecture exists between T3SS needles of different bacterial species, which could facilitate the rational design of needle-specific anti-virulence molecules that block or destabilize the T3SS machinery.

## Results and Discussion

### Identification of Key Functional Residues of PscF

**FIGURE 1:**
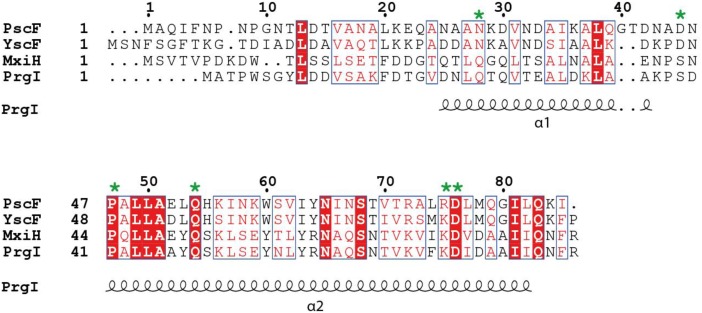
Sequence alignment of needle proteins from different bacteria. PscF from *Pseudomonas aeruginosa*, YscF from *Yersinia* sp., MxiH from *Shigella* sp., PrgI from *Salmonella* sp. The residues mutated in this study are indicated with a green asterisk. Numbers refer to the PscF sequence. Secondary structure elements from the PrgI structure are indicated underneath the sequence alignment. The figure was generated with ESPript, using the new ENDscript server (Robert and Gouet, 2014). Conserved and similar residues are shown in red and blue boxes, respectively.

**FIGURE 2:**
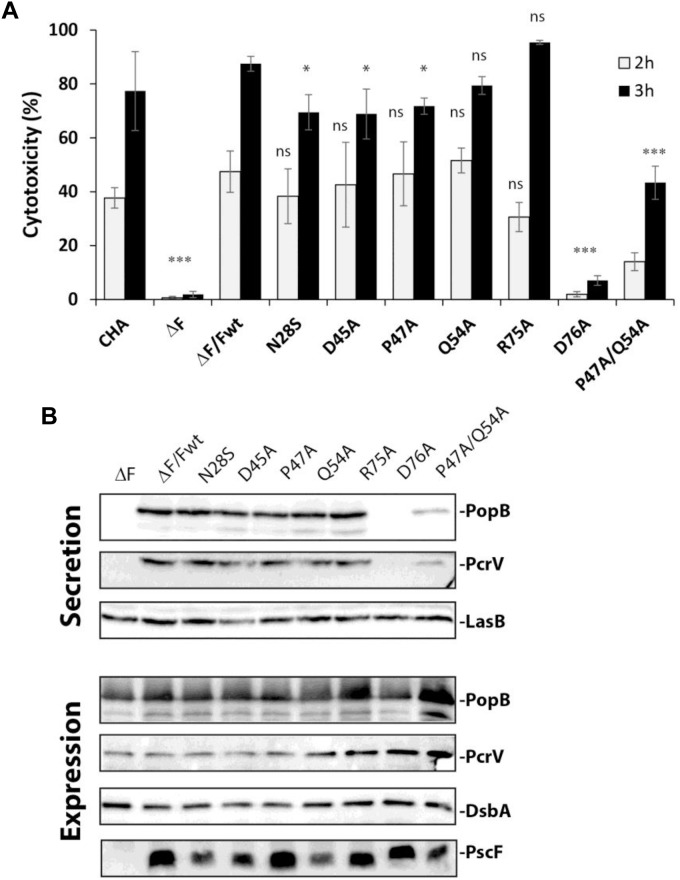
T3SS activity in *Pseudomonas* PscF mutants. **(A)** The cytotoxicity of *P. aeruginosa* strains toward macrophage cells was measured by monitoring LDH release after 2 and 3 h of infection using a multiplicity of infection (MOI) of 5. The PscF deletion mutant (ΔF), D76A, and P47A/Q54A strains show a higher statistical difference at both 2 and 3 h of infection compared to wild-type complemented strain (ΔF/Fwt). LDH measurements were corrected with values corresponding to cells that were not infected; we considered as 100% of cytotoxicity a 1% Triton X-100 treated well. Pairwise differences relative to ΔF/Fwt based on the Tukey test are indicated: ns, non-significant, ^∗∗∗^*P* < 0.001, ^∗^*P* < 0.05. Cytotoxicity of original CHA strain is also reported. **(B)** Western blot of the supernatant (secreted proteins) and total bacteria (expression) fractions after centrifugation were developed with anti-PopB and anti-PcrV antibodies. The expression of PscF variants was confirmed by loading total bacterial extracts on SDS- 15% PAGE. The Western blot was developed using anti-PscF antibodies obtained using a monomeric 6His-PscF (see section “Materials and Methods”). The D76A strain secretes almost no PopB or PcrV while P47A/Q54A secretes less of both translocators as compared to other mutants and the wild-type strain. LasB was used as loading control for the secreted protein, and DsbA as loading control for bacterial expression and as a lysis control for the supernatant (data not shown).

To uncover the structural elements that are important for the functionality of the T3SS needle in *P. aeruginosa*, we first used a mutagenesis approach coupled to phenotypic studies. We designed seven different PscF point mutants on several bases: conservation of residues between different bacterial species, results obtained in *Shigella flexneri* (Kenjale et al., 2005; Veenendaal et al., 2007), *Yersinia pestis* (Davis and Mecsas, 2007), and *Yersinia pseudotuberculosis* (Torruellas et al., 2005) and predictions/models of PscF fold (Figure 1). We generated six single mutants: N28S, D45A, P47A, Q54A, R75A, D76A, and an additional double mutant (P47A/Q54A) whose homologous residues in the *Shigella* needle protein (MxiH-P44A/Q51A) had been shown to be critical for wild-type function (Kenjale et al., 2005; Veenendaal et al., 2007). The D76A mutant had already been studied by our groups and others (Kenjale et al., 2005; Quinaud et al., 2007) and it was further characterized in this study. All mutations were introduced into the pIApG-*pscF* plasmid and transformed into a *P. aeruginosa* clinical isolate CHA strain deleted for the PscF-encoding gene (Δ*pscF*) (Pastor et al., 2005). The cytotoxicity toward macrophages of the Δ*pscF/pscF*wt complemented strain had already been reported to be the same as the original CHA strain (Quinaud et al., 2007). We then tested the ability of all complemented strains to infect macrophages measuring the release of the host cytoplasmic enzyme lactate dehydrogenase (LDH) into the supernatant. Most mutant strains killed macrophages with comparable kinetics as the wild-type strain suggesting the existence of a robust core in the protein that can accept local perturbation. However, the two strains carrying PscF-D76A or the double mutant PscF-P47A/Q54A showed significantly attenuated cytotoxicity (with *P* < 0.001) toward macrophages. The *P. aeruginosa* PscF-D76A strain killed only 7.0 ± 1.8% of all cells present in the assay while *P. aeruginosa* PscF-P47A/Q54A strain killed 43.3 ± 6.2% of cells during 3 h of infection. As a comparison, the wild-type *P. aeruginosa* strain killed 87.5 ± 2.8% of all cells during the same period (Figure 2A). Both mutations P47A and Q54A alone had no effect on cytotoxicity after 3 h of infection, in agreement with previous observations of hemolysis and invasion in *Shigella* (Kenjale et al., 2005).

To understand the cause of decreased virulence observed with the selected mutants, we first analyzed the expression of the PscF mutants and the secretion profiles of translocator proteins PopB and PcrV in those strains (Figure 2B). The T3SS was induced *in vitro* by Ca^2+^ depletion in bacterial cultures, the pellet (containing the entire cell content) and supernatant (containing secreted proteins) were separated by centrifugation and analyzed by Western blotting. All *P. aeruginosa* mutants expressed the different PscF variants (Figure 2B), demonstrating that the mutations did not affect the expression of PscF. Analysis of PopB and PcrV in secreted fractions showed reduced levels for the P47A/Q54A strain and a thin band of PopB and no PcrV for the D76A strain, results that are consistent with the decreased cytotoxicity of these two mutants in our macrophage killing assay.

Our results point out that among the conserved residues of the needle protein PscF, D76, P47 and Q54 play essential roles in T3SS functionality, in agreement with other reports on *Shigella flexneri* and *Yersinia pestis* (Kenjale et al., 2005; Torruellas et al., 2005). Interestingly in *Shigella*, the corresponding mutants (MxiH-D73A and P44A/Q51A) secrete Ipa/Ipg proteins constitutively, and are unable to invade epithelial cells and to cause hemolysis (Kenjale et al., 2005; Veenendaal et al., 2007). Other mutations like PscF-R75A in *P. aeruginosa* have no effect on cytotoxicity toward macrophages, while the homologous mutation (MxiH-K72A) in *Shigella* has a significant impact in hemolysis, suggesting a potential different mode of regulation/activation of needle protein polymerization. We thus set out to further understand the specific role of these residues in regards to: (i) proper folding of PscF, (ii) PscF’s ability to polymerize and form the needle, (iii) interaction of PscF with the tip translocator protein PcrV, (iv) secretion, localization and abundance of the PscF needle on the bacterial surface.

### *In vitro* Characterization of PscF Needle Mutants

**FIGURE 3:**
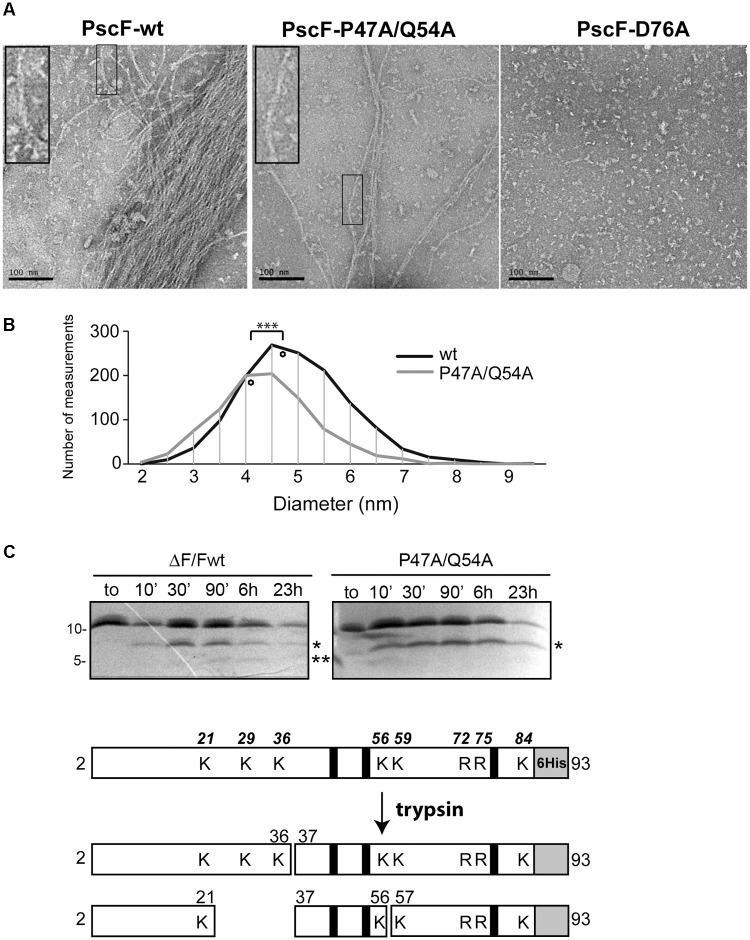
Characterization of PscF wild-type and mutant filaments. **(A)** Negative-staining electron microscopy images of purified PscF wild-type (left panel), PscF P47A/Q54A (middle panel) and PscF D76A (right panel) visualized at the same protein concentration (0.1 mg/ml). Scale bar: 100 nm. **(B)** Diameter distribution of PscF filaments measured on EM images shown in **(A)**. The diamonds represent the average diameter. Wild-type PscF has an average diameter of 4.72 ± 1.02 nm (*n* = 1390) while PscF P47A/Q54A filaments are thinner with average diameters of 4.15 ± 0.97 nm (*n* = 986). Statistical analysis: ^∗∗∗^*P* < 0.001. **(C)** Trypsin digestion profiles of purified wild-type PscF and P47A/Q54A filaments were analyzed by 16.5% Tris-Tricine and LC/ESI Mass spectrometry. Both proteins are stable with a major cleavage-band at 6.5 kDa (one asterisk) corresponding to fragments 37–93. Only for the wild-type protein is visible a second lower band of 4.5 kDa (residues 57–93) (two asterisks). A scheme of trypsin digestion kinetics determined by MS analysis is reported below, with the corresponding identified fragments. The gray box represents the 6His tag, black lines show the position of D76, P47, and Q54 residues mutated in this study. All trypsin-cleavable sites (lysines, K and arginines, R) are shown with their relative position on the top scheme.

In order to investigate the first two possibilities, i.e., that D76A and P47A/Q54A mutations could affect PscF folding and needle polymerization, we expressed and purified the two PscF mutants in *E. coli* in the absence of their two chaperones PscE and PscG. This allowed the *in vitro* production of filaments, as it was shown in the case of wild-type PscF (Quinaud et al., 2005). Purified PscF wild-type, PscF-D76A and PscF-P47A/Q54A filaments were then analyzed by negative staining electron microscopy (EM) and atomic force microscopy (AFM) (Figure 3A and Supplementary Figure S1). Both the wild-type and the P47A/Q54A PscF variants formed elongated fibers with outer diameters ranging from 2 to 10 nm indicating that the P47A/Q54A mutation does not prevent the needle polymerization process. The measurement of the diameters of both filaments on EM images suggested a tendency of P47A/Q54A PscF filaments to be slightly thinner than the wild-type ones with a statistically significant difference on diameter size distribution (*t*-test, *P* < 0.001) (Figure 3B). The diameter measurements by both EM and AFM thus indicated smaller values than the 8 nm-diameter determined by high resolution methods. This is probably due to a difficulty in identifying the borders of thin needles by EM, and the strong adsorption of the filaments on mica in the AFM experiments, as already found for cylindrical viruses (Godon et al., 2017). The D76A mutant protein could not form filaments suggesting a loss of capacity to polymerize (Figure 3A), thus explaining the lack of cytotoxicity of *P. aeruginosa* strains expressing PscF-D76A. On the other hand, in the PscF-P47A/Q54A mutant the 50% decrease in cytotoxicity could be linked to the formation of thinner fibers or could be correlated to a more subtle folding defect of the needle filament.

In order to investigate this last hypothesis we incubated both wild-type and P47A/Q54A PscF needle filaments with trypsin at room temperature and monitored protease digestion for up to 23 h. Samples were taken at different times and analyzed by SDS-PAGE and by liquid chromatography coupled to electrospray ionization mass spectrometry (LC/ESI-MS) (Figure 3C and Table 1). In our experiments, PscF filaments are a result of the polymerization of a 93-residue monomeric protein that carries a 6His-tag at the C-terminus with an expected mass of 10,218 Da (for the wild-type) and 10,135 Da (for the P47A/Q54A mutant). The SDS-PAGE patterns were apparently similar: both wild-type and P47A/Q54A proteins were surprisingly stable to trypsin digestion, and the full-length protein was also still apparent after 23 h. A major fragment at 6,500 Da (corresponding to fragment Ala37-His93) appeared on SDS-PAGE after 10 min (one asterisk in Figure 3C). The kinetics of trypsin cleavage were followed by LC/ESI-MS analysis at different time points (10, 30 min and 6 h of digestion) which allowed precise identification and relative quantification of proteolytic fragments (Table 1). All expected trypsin cleavage sites and the fragments identified by LC/ESI-MS are reported in Figure 3C. The first trypsin cleavage occurred after Lys21 and Lys36, generating the following three fragments: (Ala2-Lys21), (Ala2-Lys36) and (Ala37-His93). Subsequently, cleavage occurred after Lys56, leading to fragments (Lys37-Lys56) and (Ile57-His93). Moreover, LC/ESI-MS analyses of digested fragments highlighted interesting differences between PscF wild-type and PscF P47A/Q54A needle filaments. Indeed, an additional digested band at a lower molecular mass was visible on SDS-PAGE after 90 min for the wild-type filaments only, corresponding to specific cleavage after Lys56 (two asterisks in Figure 3C). This fragment of 4,454 Da (residues Ile57-His93) for the PscF-P47A/Q54A mutant was not visible on SDS-PAGE and detectable only by MS analysis after 6 h in the proteolysis experiment representing 1.1% of the fragment population versus 10.5% in the wild-type PscF at the same time point (Table 1). These results suggest that Lys56 is less accessible to proteolysis by trypsin in the PscF-P47A/Q54A filament compared to the PscF wild-type filament. An opposite effect was observed for Lys36, that is more accessible to trypsin cleavage in the PscF-P47A/Q54A filament (with 10.7% of fragment population corresponding to Ala37-His93 with a mass of 6,472 Da) compared to the PscF wild-type filament (with 2.5% of fragment population) (Table 1 and one asterisk in Figure 3C). Therefore, the two filaments are structurally different at least in the region around Lys36 and Lys56.

**Table 1:** Identification and relative quantifation of PscF (*wt*/mutant) proteolytic fragments by LC/ESI MS.

Proteolysis time	10 min		30 min		6 h	
Theoretical mass (Da) for tryptic fragments of PscF (wt/mutant)	PscF-WT	PscF-P47A/Q54A	PscF-WT	PscF- P47A/Q54A	PscF-WT	PscF- P47A/Q54A
	
	Measured mass (Da) *(% Area of EIC peaks*)					
2097.09 ^(1)^ (2-21)	2097.08 *(9.4%)*	2097.09 (*5.6%*)	2097.09 (*18.1%*)	2097.09 (*10.8%*)	2097.08 (*28.1%*)	2097.09 (*30.8%*)
2118.07/2035.03 ^(1)^ (37-56)	2118.07 *(1.6%)*	2035.02 (*0.2%*)	2118.07 (*3.7%*)	2035.03 (*0.3%*)	2118.07 (*17.6%*)	2035.03 (*3.5%*)
3678.86 ^(1)^ (2-36)	3678.87 *(7.7%)*	3678.86 (*13.2%*)	3678.85 (*11.4%*)	3678.85 (*12.6%*)	3678.85 (*2.9%*)	3678.85 (*3.9%*)
4454.15 ^(2)^ (57-93)	4454.36 *(2.6%)*	n.d.	4454.52 (*3.4%*)	n.d.	4454.88 (*10.5%*)	4454.18 (*1.1%*)
6555.45/6472.36 ^(2)^ (37-93)	6555.63 *(9.4%)*	6472.50 (*9.1%*)	6555.72 (*9.4%*)	6472.55 (*8.5%*)	6555.60 (*2.5%*)	6472.55 (*10.7%)*
10218.47/10135.38 ^(3)^ (2-93)	10218.67 *(69.3%)*	10135.59 (71.9%)	10218.68 (*54.0%*)	10135.46 (*67.8%*)	10218.80 (*38.5%*)	10135.46 (*49.8%)*

*Theoretical mass values for tryptic fragments of PscF (wild-type and P47A/Q54A mutant) are reported in the first column. Both PscFwt and P47A/Q54A mutant were digested with trypsin (trypsin:protein ratio 1:500 w/w) at room temperature. After 10 min, 30 min and 6 h of incubation the samples were analyzed by LC/ESI-MS. The area of the extracted ion chromatogram (EIC) peaks for each fragment were calculated and reported as a percentage of the sum of all fragment (total area of all the peaks). ^(1)^ monoisotopic mass,^(2)^ average mass, ^(3)^ mass value for the intact undigested protein, missing the methionine at N-termini; n.d., not detected.*

These results indicate that the P47A/Q54A and wild-type filaments only present a small difference on the outer surface structure (see below). Along with the fact that P47A/Q54A filaments could be slightly thinner, this could explain the decreased capacities of secretion and cytotoxicity of the strains carrying these mutations. However, one must also consider that the filaments purified form *E. coli* could be structurally distinct from needles assembled within the T3SS apparatus in *P. aeruginosa*.

### Needle Visualization on the Surface of *P. aeruginosa* PscF Mutants

**FIGURE 4:**
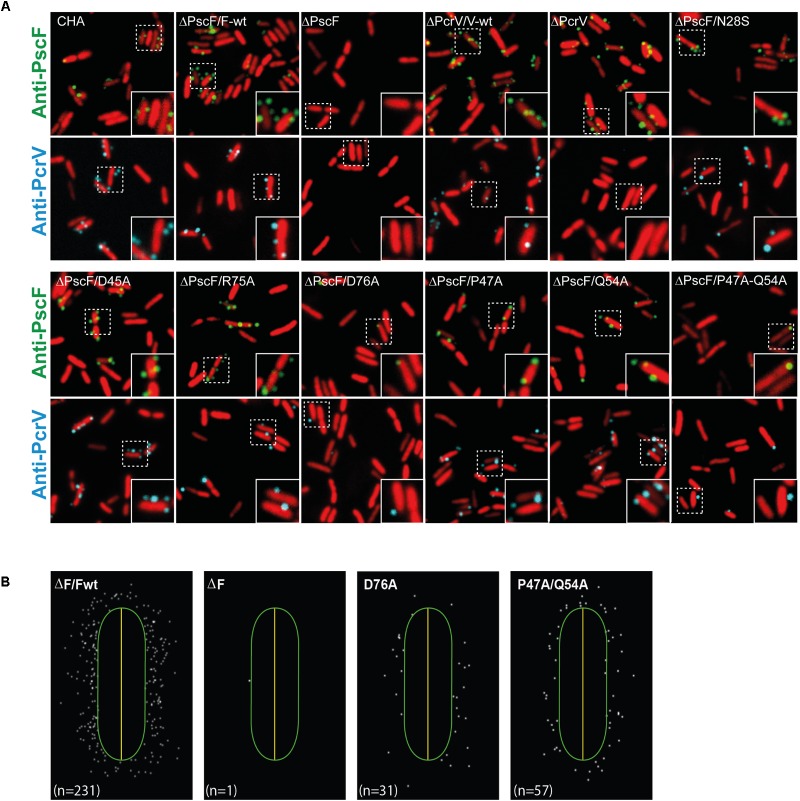
Localization of PscF-needles on the surface of *Pseudomonas aeruginosa*. **(A)** *P. aeruginosa* CHA strain isolated on cystic fibrosis patient depleted for the *pscF* gene (Δ*pscF*) and complemented with pIApG-*pscF* constructs were grown in T3SS-inducing conditions. PscF needles were visualized by immuno fluorescence on fixed bacteria using anti-PscF (in green) or anti-PcrV (in cyan). SYTO24 was used to visualize bacteria (in red). As a negative controls we used *P. aeruginosa* ΔF and ΔV, while ΔF/Fwt and ΔV/Vwt were used as a positive control. Only few PscF and PcrV spots were visible on *P. aeruginosa* strains carrying a PscF-D76A or a PscF-P47A/Q54A. **(B)** MicrobeJ reconstitution of PscF distribution around the bacteria. n = number of spots in the image.

In order to further characterize the aforementioned mutations on PscF in a more physiological context, we looked at the localization and/or abundance of the needle on the *P. aeruginosa* surface as well as its interaction with the PcrV tip protein using fluorescence microscopy. We performed immune detection of PscF on fixed cells, using antibodies generated against the PscF native needles (produced during this study) (Figure 4). Fixed cells were prepared as described in Section “Materials and Methods” and then separated into two samples: one was incubated with anti-PcrV antibodies (shown in cyan), while the other one was incubated with anti-PscF antibodies (shown in green). In both samples, DNA was stained with SYTO24 (shown in red). The Δ*pscF* and Δ*pcrV* samples were used as negative controls and presented no unspecific labeling with the anti-PcrV antibodies and just weak background with the anti-PscF used at higher concentrations. ΔPscF/PscF-wt and ΔPcrV/PcrV-wt were used as positive controls and showed clearly distinct cell surface-associated fluorescent spots for both proteins all around the bacteria (Figure 4A,B). Of note, PcrV could not be detected on the surface of the ΔPscF strain because it only assembles at the tip of the PscF needle. All PscF mutants showed similar patterns of labeling as the positive controls except for the PscF D76A- and P47A/Q54A-expressing strains that showed markedly smaller amounts of PscF and PcrV spots all around their surface (Figure 4B and Supplementary Figure S2). To eliminate the possibility that the absence of PscF on the *P. aeruginosa* surface was due to a defect in expression of PscF D76A and P47A/Q54A, we repeated the experiment with cells made permeable with Triton X-100 in order to allow the entry of the antibody into the cell. The antibodies directed toward PscF detected the protein in both permeabilized mutant strains (Supplementary Figure S3). These data are in agreement with the Western blot results and indicate that PscF mutant proteins were produced within *P. aeruginosa*. Moreover, this experiment showed that D76A and P47A/Q54A mutants have a defect in PscF export and/or assembly onto the bacterial surface.

**FIGURE 5:**
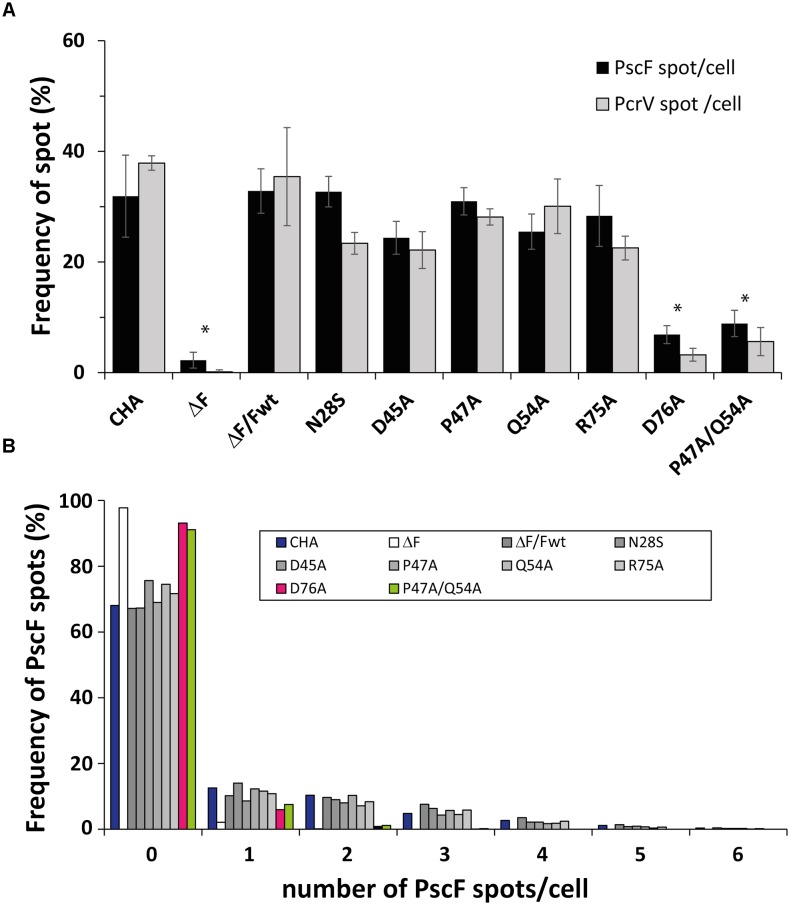
Presence of PscF and PcrV on *Pseudomonas aeruginosa* PscF-mutant strains. **(A)** Images of immunolabeled fixed bacteria in Figure 4 were analyzed with MicrobeJ to detect and associate each PscF or PcrV spot to only one bacterium. Frequency was considered as the percentage of bacteria with at least one PscF and PcrV spot on the total counted bacteria. About 30–40% of cells had needles with the PcrV tip protein on their surface, with the exception of the D76A strain with only 6.9 ± 1.6% (PscF) and 3.2 ± 1.2% (PcrV), and the P47A/Q54A strain with 8.9 ± 2.4% (PscF) and 5.6 ± 2.5% (PcrV) of cells with spots. **(B)** Distribution of the number of PscF spots/needles per bacterial cell. Between 630 and 2300 individual bacteria were counted. *P. aeruginosa* wild-type and mutant strains had between 1 to 3 PscF spots per cell, except for D76A (in pink) and P47A/Q54A (in green) that presented only one or two spots per cell. Overall comparisons using the Kruskal–Wallis’ test indicates significant differences between classes (*P* < 0.001). Pairwise differences relative to wild-type based on Dunn’s *post hoc* test are shown: ^∗^*P*<0.05.

We then quantified the PscF and PcrV spots per cell using the MicrobeJ plugin (Ducret et al., 2016) of the ImageJ program (Schneider et al., 2012), considering that one spot corresponded to one needle. Between 30 and 40% of wild-type bacterial cells showed one or more PscF spots (up to 6) per cell, with a majority of cells with 1 or 2 spots/cell (Figure 5B). Immunolabeling of bacteria expressing PscF D76A showed that only 6.9 ± 1.6% of the counted cells (*n* = 1713) presented a single visible spot and the P47A/Q54A mutant displayed 8.9 ± 2.4% of the population with 1 or 2 spots per cell (*n* = 2307). For comparison, the complemented ΔPscF/PscF-wt stain presented 32.8 ± 4.0% of cells with at least one needle (*n* = 1829) (Figure 5A,B) while the negative control ΔPscF strain presented 2.3 ± 1.4% of spots near the bacteria. In all samples, except in D76A and P47A/Q54A, the percentage of cells with PscF and PcrV spots was very similar, indicating that the mutations do not affect binding between the tip protein and the needle. In the case of D76A and P47A/Q54A only 3.2 ± 1.2% (*n* = 2126) and 5.6 ± 2.5% (*n* = 2128) of cells presented PcrV spots, respectively, suggesting that the tip/needle interaction is present, but we could not exclude that some D76A and P47A/Q54A needles do not present PcrV at their tip.

These observations explain the absence of cytotoxicity toward macrophages in the case of D76A. Interestingly, although P47A/Q54A mutations affected the number of needles detected, the synthesized needles still allowed injection of toxins, suggesting that the number of needles on the bacterial surface is less crucial than the structure of the needle *per se*. All these data strongly suggest that in *P. aeruginosa* D76A and P47A/Q54A mutants are defective in the *in vivo* needle assembly process while a concomitant instability or defect in the export of PscF through the basal body could not be excluded.

In *Yersinia pestis*, the YscF-D77A variant also shows a defect in the surface exposure of the needle whilst maintaining normal protein expression levels (Torruellas et al., 2005). In contrast, the MxiH-D73A and P44A/Q51A mutants in *Shigella flexneri* were both able to export the needle protein and assemble a NC almost normally (Kenjale et al., 2005; Veenendaal et al., 2007). Furthermore, MxiH-D73A was able to polymerize (Fujii et al., 2012) (unlike PscF-D76A) with no significant structural changes but it lacks the tip complex. Once again, this suggests that the same mutation will cause different effects on the assembly of *Shigella, Yersinia*, and *Pseudomonas* T3SS needles.

### Modeling of the *P. aeruginosa* PscF Needle

**FIGURE 6:**
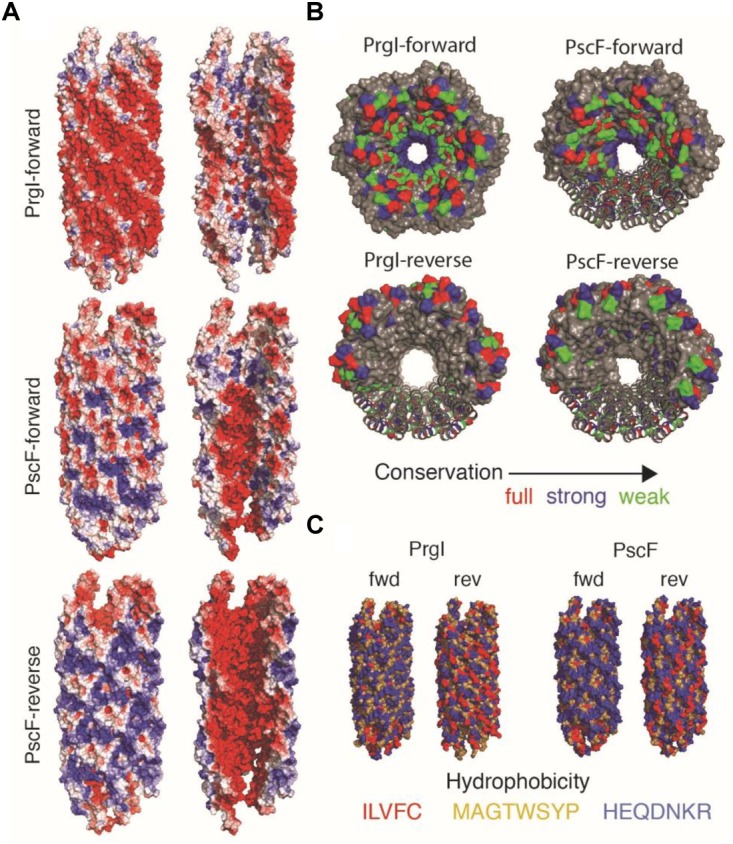
Needle reconstruction for PscF wild-type and mutants. **(A)** The PrgI Solid-state NMR structure was used as a model for the construction of PscF in the Forward and Reverse orientations. Mutants D76A and P74A/Q54A were constructed on the PscF-forward model. The exterior view (left) and lumen view (right) of the T3SS needle models are colored by surface electrostatic potentials, at pH 7, using the APBS plugin for PyMol. **(B)** Distribution of conserved residues distribution on PrgI and PscF needle models in Forward and Reverse orientations. **(C)** Hydrophobicity distribution of the different models shown in **(B)**.

In order to understand at the molecular level the impact of the D76A and P47A/Q54A mutations, we performed molecular modeling of the *P. aeruginosa* PscF needle, taking advantage of the solid-state NMR structure of the *Salmonella* PrgI needle (Loquet et al., 2012). We constructed two different models for the PscF needle: one using the protein in the same orientation as PrgI in the filament (i.e., with the C-termini inside and the N-termini outside the needle; that model will be named PscF-forward); and the other one supposing that the PscF protomer is inverted with the N-termini residue inside and the C-termini residue outside the needle structure (PscF-reverse) (Figure 6). Optimal models were subjected to a brief structural minimization protocol using the Chimera molecular modeling/visualization package (Pettersen et al., 2004) (default parameters) to remove atomic clashes (VDW overlap). Pre- and post-minimization structures were analyzed using the PSVS webserver (Bhattacharya et al., 2007) to evaluate the structural improvements in the monomeric models. After minimization, all homology models showed significant improvements in both clash scores and favorable Ramachandran angles. The energetically minimized structures were then used to reconstruct PrgI-like T3SS needles, and were built up from 29 monomers to form the needle filaments. To compare the two models of PscF to the needle architecture in *Salmonella* we also constructed a PrgI-inverted needle (PrgI-reverse).

The three resulting needle architectures: PscF-reverse, PscF-forward and PrgI-reverse are shown in Figure 6. We then tried to understand if one of the two PscF needle models (PscF-reverse or PscF-forward) was more favorable. To do so, we computed several parameters related to the global architecture: surface electrostatic potential (Figure 6A), position of conserved residues (Figure 6B) and hydrophobicity (Figure 6C) and compared them to the experimental solid-state NMR *Salmonella* PrgI needle structure. Surprisingly none of the PscF needle models showed the same electrostatic distribution as the experimental PrgI needle structure (top in Figure 6A), displaying an astonishing pattern of circular succession of negative/positive charges at the lumen surface, while the external surface was predominantly negatively charged. Both PscF needle models showed a strong negative charge distribution at the inner surface, while the external surfaces were much more positively charged compared to PrgI needles. In addition, no particular succession of positively/negatively charged patterns could be seen for either PscF model. In PrgI needles, this lumen pattern arises mostly from D70 and Q77 (negative) and K66 and R80 (positive). Equivalent amino acid positions in PscF D76, Q83, and R72 are conserved (Figure 1, 6B); however, R80, the last residue in PrgI, is absent from PscF, leading to a pronounced change in the electrostatic charge distribution at the lumen surface.

Next, we investigated the hydrophobic surface of the models (Figure 6C) considering that the PrgI needle is characterized by a strong network of hydrophobic interactions at the lateral interfaces between subunit i and i+5/6 (Loquet et al., 2012). PscF-reverse and PrgI-reverse models both exhibit an unfavorable hydrophobic external surface while the PscF-forward model shows a favorable hydrophilic surface, strengthening the energetic relevance of the PscF-forward model. Finally, we examined the distribution of the conserved residues on the different needle models (Figure 6B). The PrgI needle structure has been defined by a typical distribution of conserved residues among the T3SS needle subunits, with almost all conserved residues pointing inside the needle pore while the external surface is made of non-conserved amino acids. Visualization of these conserved needle residues on the PscF models lead to two distinct situations: PscF-forward presents many conserved residues at the lumen surface, while this lumen surface in the PscF-reverse model is mostly composed of non-conserved residues. It has been suggested that the presence of non-conserved residues exposed to the external surface of PrgI could provide a way to evade immune response of the host cell (Loquet et al., 2012). The same distribution is observed for the PscF-forward model, and together with our observations on the hydrophobic and electrostatic surfaces it provides a substantial hint toward the relevance of the PscF-forward model compared to the PscF-reverse model.

**FIGURE 7:**
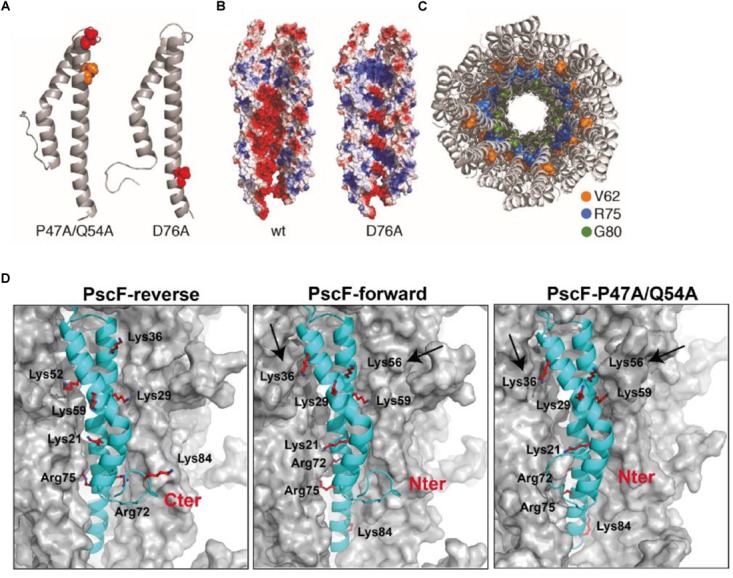
Model of the PscF P47A/Q54A and PscF D76 assembled filament. **(A)** Ribbon representation of PscF monomer carrying the two mutations: D76A and P47A/Q54A. **(B)** Electrostatic surface representation of the two mutant reconstituted filaments with a channel view. **(C)** Top view of a PscF-forward wild-type model, colors show residues implicated in binding of the T3SS-inhibitor phenoxyacetamide (Bowlin et al., 2014). **(D)** Comparison between reverse and forward PscF and PscF-P47A/Q54A filaments. PscF-forward model (with the N-termini exposed toward the outside), with Lys36, Lys29, and Lys21 solvent-exposed, Lys56 and Lys59 less accessible, and Lys84, Arg72, and Arg75 completely buried. PscF-reverse model (with the C-termini exposed toward the outside), with solvent accessible residues: Lys84, Arg75, Lys59, and Lys36. The PscF-P47A/Q54A forward model showing accessibility of the same residues as compared to the PscF-forward wild-type model except for the Lys56 and Lys36 that are slightly less or more trypsin-accessible, respectively (indicated by two arrows). All needle models are represented as surfaces with one monomer of PscF represented as a cyan ribbon. All trypsin-cleavable sites are shown as sticks.

Subsequently, we constructed PscF needle models for the two mutants: D76A and P47A/Q54A (Figure 7B), adopting the forward model orientation. In the D76A model, the distribution of the charges is drastically affected by the point mutation; D76 is located in the central hollow section of the needle and the mutation into alanine modifies the overall negative charge distribution, which could be an explanation to the lack of polymerization *in vitro* of this mutant. The P47A/Q54A model, however, suggests that the mutated residues are buried in the subunit–subunit interfaces and the overall charges are more similar to the wild-type PscF-forward model. According to the atomic depth measurements (Chen and Pellequer, 2013), the regions 45–47 is the deepest, farthest away from the solvent, in the whole needle. Any change in deeply buried residues is expected to impact proteins stability (Chakravarty and Varadarajan, 1999), that could explain the significant difference in the number of needle on bacteria surface. Considering the equivalent amino acids in PrgI (i.e., P41 and Q48), we examined the residues involved in the subunit–subunit interfaces by taking into account the solid-state NMR restraints used for the PrgI needle structure determination (see Figure 2 in Loquet et al., 2012). P41 in PrgI is involved in strong contacts between subunit i to i+11 forming the so-called axial interface, while Q48 is weakly involved in the intramolecular fold. This suggests that the double P47A/Q54A mutation in PscF could lead just to a moderate perturbation of the needle arrangement that could explain why this mutant still polymerizes *in vitro*.

We then used the data obtained from trypsin proteolysis and MS analysis to explore the structural differences between wild-type and mutant P47A/Q54A filaments and to validate our model. As mentioned above, we monitored trypsin digestion of both fibers by LC/ESI-MS (Figure 3C and Table 1). This experiment allowed us to conclude that the residues that were the most accessible to trypsin digestion, and thus were solvent exposed are: Lys36, Lys21, and Lys56 (and probably Lys29, absent in MS digested-fragments). Interestingly, these experimental results are only in agreement with the PscF-forward wild-type model (with the N-termini exposed toward the outside), where Lys36, Lys29, and Lys21 are expected to be the most exposed to the solvent, Lys56 and Lys59 are less accessible, and Lys84, Arg72 and Arg75 are completely buried (middle in Figure 7D). On the other hand, in the PscF-reverse wild-type model (with the C-termini exposed toward the outside), the residues that would be solvent accessible are: Lys84, Arg75, Lys59, and Lys36 (left in Figure 7D). Lys84, in particular, is located just before the C-terminus. In this configuration, the C-terminal 6His-tag would have been expected to be quickly cleaved by trypsin, but no fragment corresponding to this cleavage was observed for any of the peptides analyzed by LC/ESI-MS (Table 1). Moreover, the PscF-forward P47A/Q54A model with Lys56 less solvent-exposed and Lys36 more solvent-exposed (right in Figure 7D) is also in agreement with our trypsin digestion results. The digestion-fragment at 4,454 Da (corresponding to a cleavage after Lys56) was less abundant in the mutant compared to the wild-type (1.1 vs. 10.5%), while the digestion fragment corresponding to a cleavage after Lys36 was more abundant in the mutant compared to the wild-type (10.7 vs. 2.5%) as discussed above (Figure 3C and Table 1).

Thus, based on our biochemical, structural and molecular modeling data, we hypothesize that the T3SS needle of *P. aeruginosa* follows the PscF-forward model with the N-terminus pointing toward the outside of the fiber and the C-terminus consequently hidden inside the needle’s hollow cavity. Our results imply that the overall architecture of the needle is therefore conserved between *Pseudomonas*, *Salmonella*, and *Shigella* spp., highlighting the importance of the orientation of the small 9-kDa protein inside the oligomeric structure. D76A and P47A/Q54A mutations are located at the C-terminal helix, which is well-conserved in the sequence among bacterial species. Their effects on needle polymerization and stability as well as their importance for functional regulation of needle assembly as shown by our analyses of electrostatic charges present at the lumen surface point to an important role of the C-terminal helix in the assembly mechanism. The impact of the mutations D76A and P47A/Q54A, and their homologs in T3SS needles of other bacteria, suggests that regions facing the inner cavity or deeply buried within the needle play a significant role in needle stability and/or function. It also suggests that local changes of the sequence of the subunit protein in crucial regions, as exemplified here for the two mutants and in previous studies, might lead to important perturbations on different aspects of the needle structure-function interplay, on both a structural (e.g., structural instability, surface electrostatic distribution) and functional basis. Moreover, the charge distribution in the lumen may be critical in needle assembly as suggest by the defect in polymerization in the D76A variant and the previous observation that the PscF-D76A protein overexpressed in *P. aeruginosa* CHA strain has a dominant-negative effect on assembly of the wild-type PscF needle (Quinaud et al., 2007).

### Understanding the Mode of Action of T3SS Inhibitors

To go further we used our proposed *P. aeruginosa* T3SS needle model to understand the mode of action of T3SS-inhibitors, such as the phenoxyacetamide family, that was hypothesized to act inside the cavity of PscF (Bowlin et al., 2014). In this work, the authors suggested that PscF residues V62, R75 and G80 were involved in direct binding of phenoxyacetamide. In our model, only G80 is exposed to the lumen surface (Figure 7C in green), while V62 (in orange) and R75 (in blue) are located at subunit interfaces. These observations suggest that the three amino acid positions, upon binding with phenoxyacetamide, may decrease needle stability (for V62 and R75); in addition, it is also possible that binding could change the surface of the lumen (G80). These three residues appear to be close enough in our PscF needle model to speculate the presence of a single binding site, as suggested by the Moir group (Bowlin et al., 2014). We therefore suggest that it is possible to design other molecules that interact with multiple residues starting from the lumen structure model in order to block T3SS action.

The T3SS needle represents an excellent and promising molecular target in the fight against Gram-negative pathogens, since it is easily accessible to small molecules that do not need to cross bacterial membranes, and is non-essential for survival. Thus, small molecules affecting its function could block virulence without eliciting high levels of antibiotic resistance.

Our work provides a new model for the *P. aeruginosa* PscF-needle, that was validated *in vitro* by experimental data combining mutagenesis, proteolysis, and Mass Spectrometry experiments. Moreover, the phenotypic characterization of PscF mutants *in vivo* showed that the majority of the single mutations introduced in the needle have no effect on T3SS-toxicity and are not sufficient to disrupt needle polymerization, with the exception of D76A. This underlines the importance of the charge in the face of the lumen for assembly of a functional needle.

A detailed molecular comprehension of the structure–function relationship of the T3SS needle should advance future developments of anti-virulence molecules.

## Materials and Methods

### Bacterial Strains and Culture Conditions for T3SS Expression

Strains and plasmids used in this study are presented in Supplementary Table S2. Cytotoxic *P. aeruginosa* cystic fibrosis isolate CHA carrying appropriate chromosomal deletions of *pscF* gene (Δ*pscF)* was transformed with plasmid pIApG-*pscF* wild-type or mutants that expressed *pscF* with a T3SS inducible promoter. Cultures were grown overnight in Luria-Bertani (LB) at 37°C at 300 rpm in the presence of 300 μg/ml of carbenicillin. Then next day, they were diluted to an optical density measured at 600 nm (OD_600_) of 0.1 A.U. in LB in T3SS-inducing conditions by adding 5 mM EGTA and 20 mM MgCl_2_ (Pastor et al., 2005; Dasgupta et al., 2006). When the OD_600_ reached 1.0 A.U., cells were centrifuged, and then the pellet (entire bacterial protein content) and the supernatant (the secreted protein fraction) was analyzed by Western blotting. Anti-PopB and anti-PcrV antibodies (Goure et al., 2004) were diluted to 1:10,000 and 1:3,000, respectively. Anti-PscF antibodies were raised in rabbits (Covalab, France) using monomeric 6His-PscF (Quinaud et al., 2005). The antibodies were further purified on Protein-A column and diluted 1:500 for Western blot analysis. As a loading control we used LasB (a secreted protein) and DsbA (a periplasmic protein) diluted to 1:2,000 and 1:10,000, respectively. Lysis control was done on secreted protein fractions using DsbA antibodies. All mutants were produced using the Quick-Change site-directed mutagenesis II kit (Stragatene). Primers are listed in Supplementary Table S2.

### Cytotoxicity Assay

Lactate dehydrogenase release into the supernatant was measured using the Cytotoxicity Detection Kit by Roche Applied Science, following the recommended protocol. Briefly, J774 cells were seeded at 2 × 10^5^ in 48-well plates the day prior to the experiment, and infected in DMEM medium at MOI of 5 with *P. aeruginosa*Δ*pscF*-pIApG-*pscF* constructs at OD_600_ = 1 A.U. After 2 and 3 h, 30 μL of cell supernatants were mixed with 100 μl of reaction mix and the OD was read at 490 nm. OD values were subtracted from that of uninfected cells. The 100% cell death value was quantified after addition of 1% Triton X-100, in duplicates. The experiment was performed in triplicate wells (except for the P47A/Q54A mutant for which six replicates were performed). Results represent the means and standard deviations of the triplicates. Statistics were calculated using SigmaPlot software. For multiple comparisons, a one-way analysis of variance (ANOVA) test was performed, followed by Tukey’s test for pairwise comparisons.

### Immunofluorescence

Two different *P. aeruginosa* genetic backgrounds were used*:* CHAΔ*pcrV* complemented or not with pIApG-*pcrV*-wt, and CHAΔ*pscF* complemented with all pIApG-*pscF* constructs or with the wild-type *pscF*. Cells were grown under T3SS-inducing conditions up to an optical density measured at 600 nm (OD_600_) of 1 A.U. and then culture was fixed with 4% PFA in 25 mM HEPES pH 7.4 overnight at 4°C. After centrifugation at 6,000 rpm for 15 min the pellet was washed three times with 1 ml PBS, then incubated for 30 min at RT in 1 ml PBS with 0.5% BSA (BS, Blocking Solution). Then cells were divided into two Eppendorf tubes and spin down, each pellet was resuspended and incubated for 30 min at room temperature in 50 μl of BS containing of anti-PcrV (1:200) (Goure et al., 2004) or 50 μl BS with anti-PscF (1:50). Anti-PscF antibodies were produced in rabbits (Biotem) from the PscF fibers purified under native conditions from *E. coli* BL21 (DE3). After three washing steps with 1 ml PBS, the pellet was resuspended into 50 μl of BS containing anti-rabbit coupled to Cy3 (Jackson ImmunoResearch Laboratories) with a final dilution of (1:500) and SYTO24-Green (Life Technologies) with a final dilution of (1:2000) and further incubated 30 min at RT. After three final washes, the cells were resuspended into BS. One drop of each sample was deposited on a 8-chambered labteck (Thermo Fisher Scientific) and liquid 1.5% low-melting agarose at 37°C or 1.5% agar pad was added on the top of the drop and visualized on a IX71 Olympus microscope controlled by the CellR Olympus system and driven by Xcellence software (Olympus). Images were captured with a Hammamatsu Orca-ER camera using a 100x (N.A. 1,30) oil objective.

To investigate for the presence of PscF inside the cells in PscF-mutants, cells were fixed with 4% PFA, washed as previously described, then permeabilized with 0.25% Triton X-100 for 5 min at room temperature to allow the anti-PscF to enter cells (method adapted from Cimino et al., 2006). Triton X-100 was then washed out three times with 1 ml PBS before continuing the protocol as described above from BS incubation.

### Quantification of Fluorescent Spots Associated With Bacteria

Quantification was done using the MicrobeJ plugin of ImageJ (Ducret et al., 2016)^1^ and^2^. Briefly, for bacterial identification we used the same setting (area, length, width, curvature of bacteria) for all images. For PcrV and PscF spot analyses a Tolerance of 100 was used, and only in the case of PscF a filter of intensity between 250-max was added in order to eliminate background noise and unrelated spots. For the final step of analyses by MicrobeJ, namely the association between each identified bacterium and the spots, we used two filters: inside and outside with “exclusive” settings, in order to associate each spot (that could be inside or outside the bacteria) with just one bacterium. Averages were calculated from the results of 5 to 11 independent images taken for each mutant. The frequency was considered as being the percentage of cells with at least one spot.

Statistics were calculated using SigmaPlot. For multiple comparisons, a one-way analysis of variance (ANOVA) test was performed, followed by Dunn’s test.

### PscF Expression and Purification and Mutant Construction

*Escherichia coli* BL21 (DE3) cells were transformed with pET22b-PscF (wild-type, D76A and P47A/Q54A) that generated a protein with a 6His-tag at the C terminus (Quinaud et al., 2005). Cells were grown in LB media supplemented with 100 μg/ml ampicillin at 37°C, under agitation (200 rpm) until they reached an OD_600_ = 0.7–0.8 A.U. Protein expression was induced by addition of 1 mM IPTG and growth was continued for up to 3 h, then cells were centrifuged for 30 min at 5,500 rpm at 4°C. Pellets were resuspended at 4°C in 25 ml of lysis buffer (25 mM Tris-HCl pH 8.0, 200 mM NaCl, 25 mM Imidazole) containing benzonase. Cells were lysed at 18 kPsi in a cell disrupter and centrifuged for 30 min at 15,000 rpm at 4°C. The soluble fraction was applied on a 1 ml resin Ni-NTA superflow (QIAGEN) in a batch column. The column was washed with lysis buffer and eluted the same buffer containing 200 mM imidazole. Sample purity was checked by SDS-PAGE and Mass Spectrometry. Mutants were produced using the Quick-Change site-directed mutagenesis II kit (Stragatene) and verified by DNA sequencing. Primers are listed in the Supplementary Table S2.

### Trypsin Digestion

PscF-wt and PscF-P47A/Q54A proteins were purified as described above. Trypsin digestion was carried out according to the following procedure: trypsin was dissolved into a buffer containing 25 mM Tris-HCl pH 8.0, 200 mM NaCl and digestion was performed using a (trypsin:protein) ratio of 1:500 (w/w). The reaction was performed at RT and samples were collected at different time points (10 min, 30 min, 1 h 30 min, 6 h, ON) and analyzed by 16.5% Tris-Tricine gel and by LC/ESI-MS.

### Electron Microscopy

Four μl of the protein samples were absorbed to the clean side of a carbon film on mica, stained with sodium silico tungstate and transferred to a 400-mesh copper grid. The images were taken under low dose conditions (<10 e^−^/Å^2^) at a magnification of 23,000 and 49,000 times with defocus values between 1.2 and 2.5 μm on a Tecnai 12 LaB6 electron microscope at 120 kV accelerating voltage using a CCD Camera Gatan Orius 1000. Size determination of the PscF filaments was performed in Gwyddion (Nečas and Klapetek, 2012) using the raw.dm3 EM files, measurements were done at different positions along the filaments using the “Measure distance” tool. Additional measurements were performed in ImageJ (Schneider et al., 2012). Statistics were calculated using SigmaPlot. For single comparisons, a Mann–Whitney Rank Sum Test was used.

### Atomic Force Microscopy

A 2.5 μl drop of reconstituted filaments in deionized water (dilution 1/5000) was deposited on freshly cleaved mica, incubated for 3 min, washed with 1 ml of water with 80 μl drop steps to remove excessive salt crystals, and finally dried with nitrogen gas. Imaging was performed on a Multimode 8, Nanoscope V (Bruker) equipped with NanoScope software (Bruker, Santa Barbara, CA, United States). Imaging was done with peak force imaging mode at ∼1 Hz rate, with 512 or 1024 pixel sampling and other parameters were adjusted automatically with ScanAsyst mode in air. A ScanAsyst-air (Bruker) cantilever with nominal 2 nm tip radius, 70 kHz frequency and 0.4 N/m spring constant was used. Images were processed with Gwyddion (Nečas and Klapetek, 2012), and if needed stripe noise was removed using DeStripe (Chen and Pellequer, 2011). Size determination of the filaments was performed in Gwyddion by using manual cross-sections (3px thick) and by measuring their maximum heights. Average values and standard deviations are reported. The computation of atomic depth was performed using the Adepth server (Chen and Pellequer, 2013).

### LC/ESI Mass Spectrometry

Liquid Chromatography Electrospray Ionization Mass Spectrometry (LC/ESI-MS) was performed using a 6210 LC/ESI-TOF mass spectrometer interfaced with an HPLC binary pump system (Agilent Technologies). The mass spectrometer was calibrated in the mass-to-charge (*m/z*) 300–3200 range with standard calibrants (ESI-L, Low concentration tuning mix, Agilent Technologies) before measurements and mass spectra were recorded in the 300–3200 *m/z* range. MS acquisition was carried out in the positive ion mode with spectra in the profile mode. The MS instrument was operated with the following experimental settings: the ESI source temperature was set at 300°C; nitrogen was used as drying gas (7 l/min) and as nebulizer gas (10 psi); the capillary needle voltage was set at 4000 V. Spectra acquisition rate was of 1.03 spectra/s. All solvents used were HPLC grade (Chromasolv, Sigma-Aldrich), trifluoroacetic acid (TFA) was from Acros Organics (puriss., p.a.). Solvent A was 0.03% TFA in water, solvent B was 95% acetonitrile-5% water-0.03% TFA. The MS spectra were acquired and the data processed with MassHunter workstation software (*v.* B.02.00, Agilent Technologies) and with GPMAW software (v. 7.00b2, Lighthouse Data, Denmark).

Just before analysis each trypsin digested protein was diluted with solvent A to a final concentration of 5 μM and thermostatted at 10°C in the autosampler; 8 μl of each sample were injected. Samples were first trapped and desalted on a reverse phase-C8 cartridge (Zorbax 300SB-C8, 5 μm, 300 μm ID × 5 mm, Agilent Technologies) for 3 min at a flow rate of 50 μl/min with 100% solvent A and then eluted and separated onto a RP-C8 column (Jupiter, 5 μm, 300 Å, 1 mm ID × 50 mm, Phenomenex) at a flow rate of 50 μl/min using the following linear gradient: from 5 to 95% solvent B in 15 min, then remaining 2 min at 100% solvent B and finally re-equilibrating the column at 5% solvent B for 10 min.

### Modeling

Optimal models of PscF-forward, PscF-reverse and PrgI-reverse protomers were built based on the PrgI protomer structure of the *Salmonella* needle structure by extracting chain A from the 2LPZ pdb file. MODELLER (Eswar et al., 2007), version 9.16, was used to individually align the forward (N-C:N-C) and reverse (N-C:C-N) PscF sequences to PrgI as well as a reverse (N-C:C-N) variant of PrgI for comparison (align2d.py). Initial single-chain models for the D76A and P47A/Q54A were prepared in the same way. The resulting alignment files and the PrgI subunit model were used as additional input to MODELLER to construct subunit models with secondary structure corresponding to that of PrgI (model-single.py). Ten models were calculated in each case and an overall assessment was manually carried out, judging energetic qualities and alignment to PrgI, to derive single optimal models for all cases. In the case of both forward and reverse PscF models, C-terminal helices were additionally enforced by alteration of the Phi and Psi backbone angles with PyMOL. Optimal models were subjected to a brief structural minimization protocol using the Chimera molecular modeling/visualization package (Pettersen et al., 2004) (default parameters) to remove atomic clashes (VDW overlap). PrgI-like T3SS needles were reconstructed in PyMol by aligning the constructed subunit models, by secondary structure, to the 29 chains of the PrgI (PDB code: 2LPZ) needle model.

The complete 29-chain needle structures, for wild-type (forward and reverse) and mutated subunits, were refined using a cluster-based installation of Rosetta (Das and Baker, 2008) (relax.mpi.linuxgccrelease – relax:fast), wherein the backbone heavy atoms were fixed in space but the positions of side-chains were allowed to evolve with respect to energetic minimization. The best resulting structures (as determined by overall Rosetta energy), calculated from a pool of 3 were then submitted to the MolProbity webserver (Davis et al., 2007) in a protonated form for validation (Supplementary Table S1).

## Data Availability

Publicly available datasets were analyzed in this study. This data can be found here: https://www.nature.com/articles/nature11079.

## Author Contributions

VJ, IA, and AL designed the study and assembled the results. CL performed protein purifications and sample preparations for biochemical, EM and AFM experiments. DF and J-MT performed the EM and AFM experiments, respectively. J-LP analyzed and interpreted both images. All model building, computer minimizations and structure interpretations were performed by JT, with the supervision of AL and BH. CG constructed plasmids and mutants and perform initial cytotoxicity assays under the supervision of IA and EF. Macrophages cultures were prepared by SB. VJ performed cytotoxicity, florescence microscopy and biochemical experiments, with data analysis and interpretation. SB and DL contributed to the microscopy experiments and ImageJ/MicrobeJ analysis, while JB helped with Western blots. LS performed mass spectrometry experiments, data analysis, and interpretation. VJ, AD, and AL wrote the paper, with input from all authors.

## Conflict of Interest Statement

The authors declare that the research was conducted in the absence of any commercial or financial relationships that could be construed as a potential conflict of interest.

## Footnotes

1 https://imagej.nih.gov/ij/

2 http://www.microbej.com/
