# Supplementary figures and images for: Structural and Functional Characterization of the Type Three Secretion System (T3SS) Needle of Pseudomonas aeruginosa
Source: Front Microbiol. 2019 Mar 29;10:573. doi: 10.3389/fmicb.2019.00573 (PMC6455054; doi:10.3389/fmicb.2019.00573)

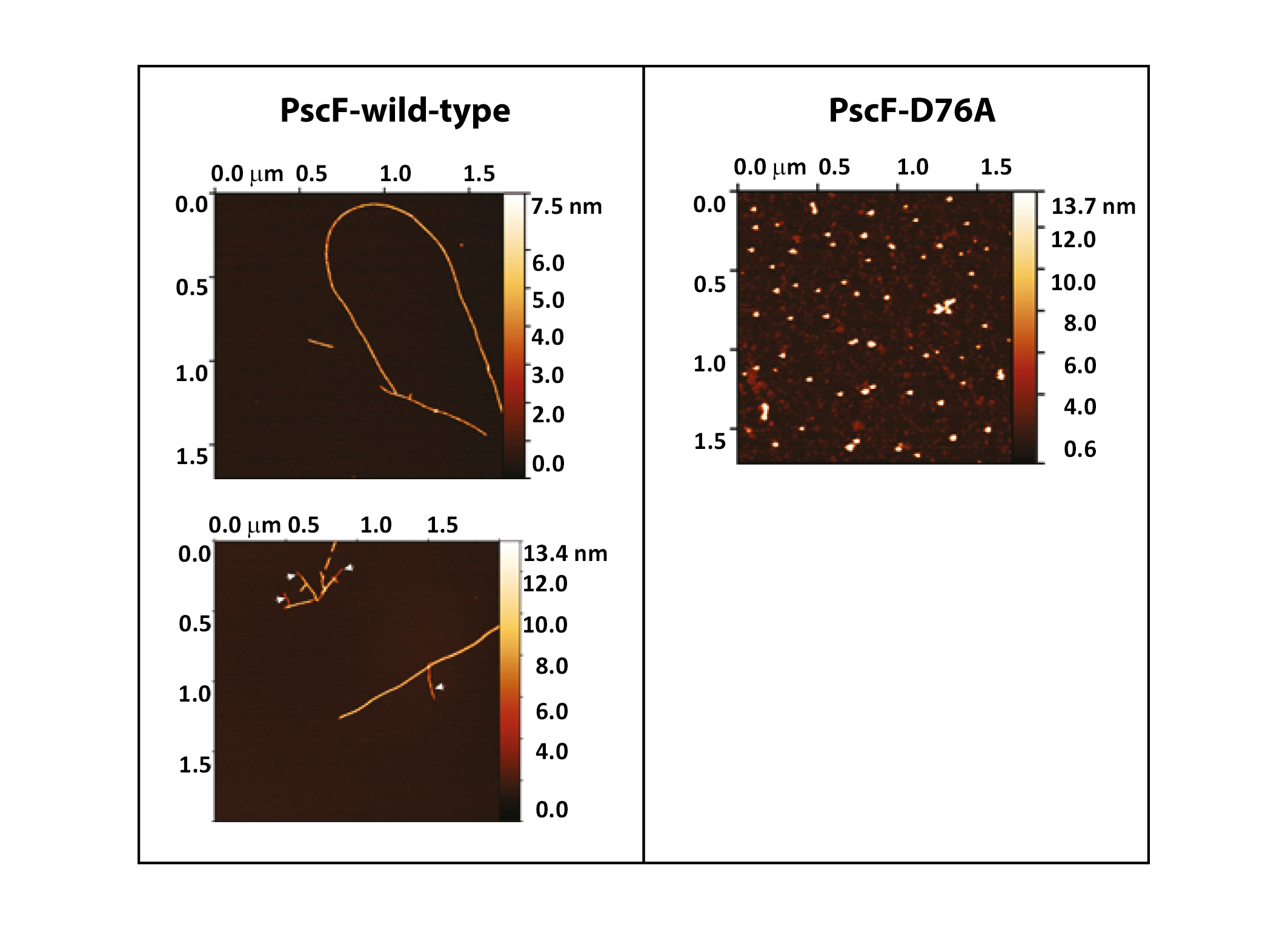

Supplement: Supplementary file 1 [file Image_1.tif]

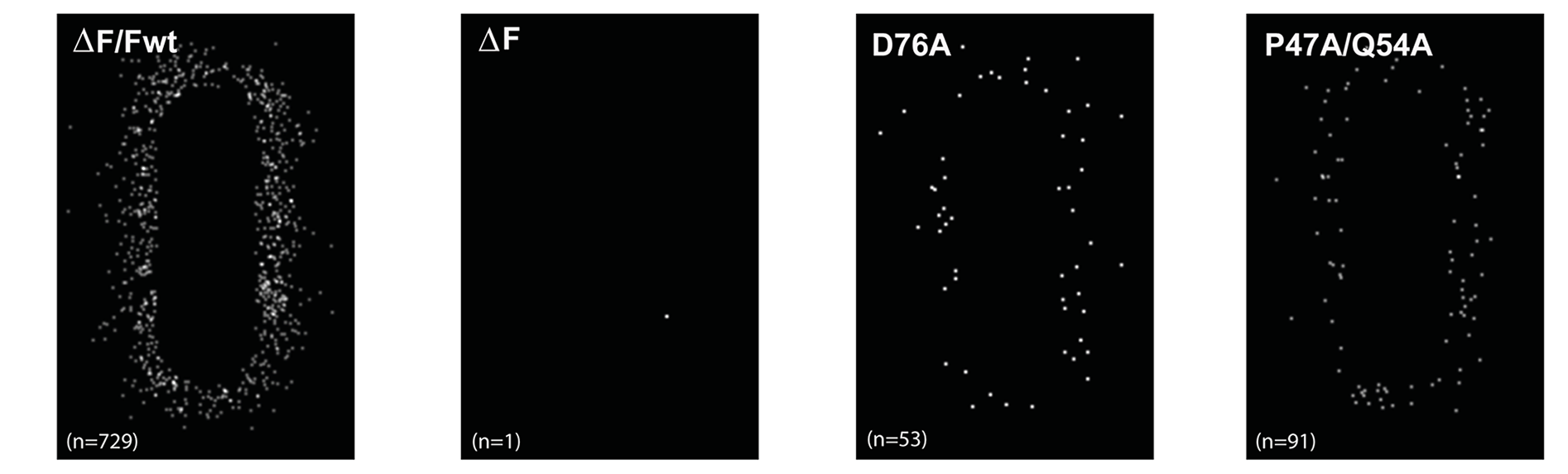

Supplement: Supplementary file 2 [file Image_2.tif]

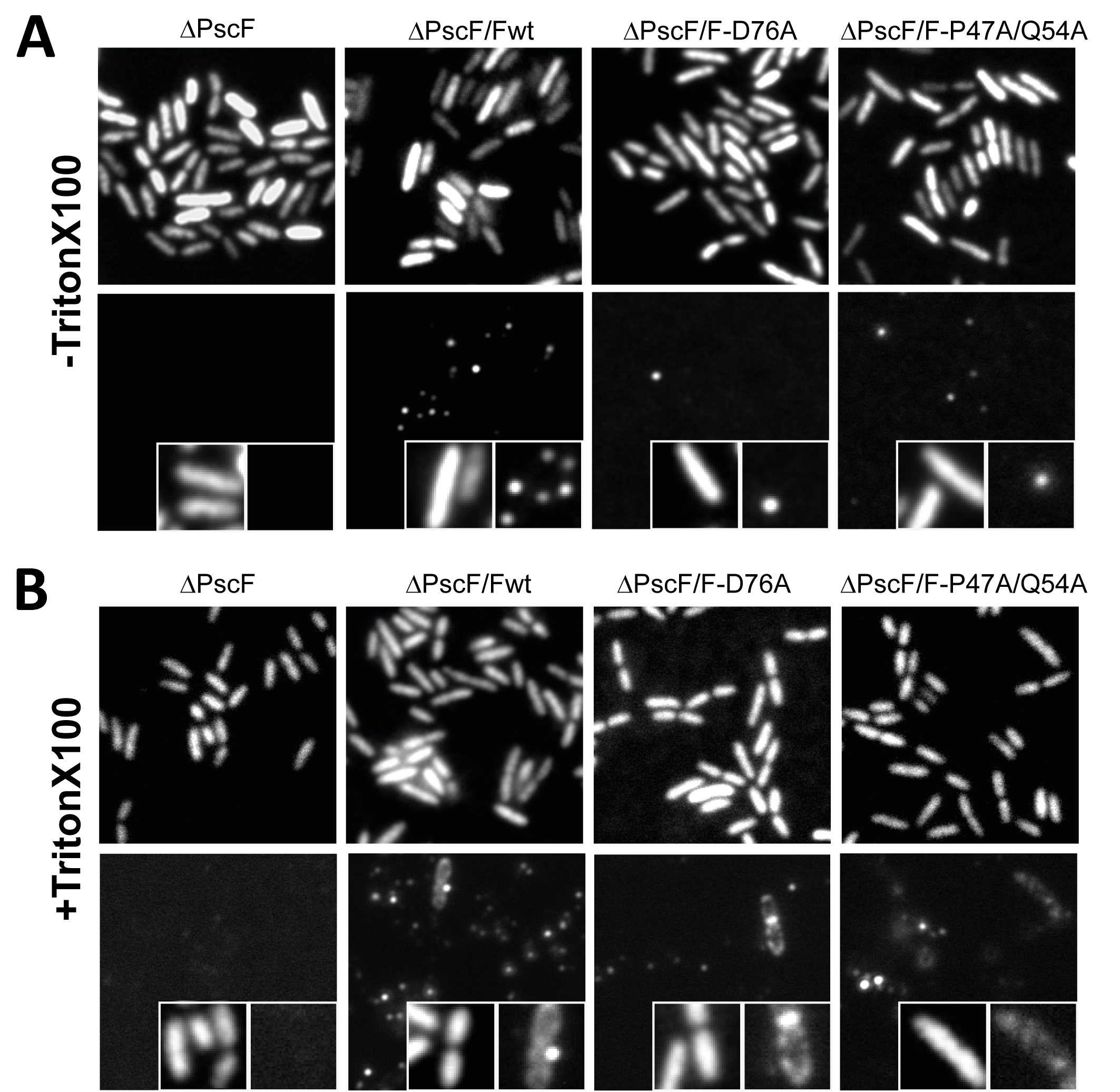

Supplement: Supplementary file 3 [file Image_3.tif]
