# Supplementary material for: Structural and Functional Characterization of the Type Three Secretion System (T3SS) Needle of Pseudomonas aeruginosa
Source: Front Microbiol. 2019 Mar 29;10:573. doi: 10.3389/fmicb.2019.00573 (PMC6455054; doi:10.3389/fmicb.2019.00573)
Supplement: Supplementary file 4 [file Table_1.docx]

**TABLE S1:** A subset of the structural validation scores for the homology models before and after Chimera minimization. Significant improvements in clash score (overall and van der Waals) are observed for all models (best = zero). Scores calculated by the PSVS webserver.

| Subunit model | Chimera  minimization | MolProbity  Clash score | Ramachandran favored+allowed | Avg. VdW clashes |
| --- | --- | --- | --- | --- |
| PrgI  (2LPZ:A) | From PDB | 1.61 | 100% | 0.05 |
| PrgI  (reverse aligned) | pre | 86.29 | 97.4% | 2.68 |
|  | **post** | **12.10** | **96.2%** | **0.28** |
| PscF  (aligned) | pre | 79.44 | 97.6% | 2.46 |
|  | **post** | **5.94** | **100%** | **0.18** |
| PscF  (reverse aligned) | pre | 116.56 | 94.1% | 3.61 |
|  | **post** | **7.42** | **98.8%** | **0.23** |
