# Supplementary material for: Structural and Functional Characterization of the Type Three Secretion System (T3SS) Needle of Pseudomonas aeruginosa
Source: Front Microbiol. 2019 Mar 29;10:573. doi: 10.3389/fmicb.2019.00573 (PMC6455054; doi:10.3389/fmicb.2019.00573)
Supplement: Supplementary file 5 [file Table_2.docx]

**TABLE S2: Summary of strains, plasmids and primers used in this study.** Underlined nucleotides indicate the mutated amino acid.

| **STRAINS** | **Characteristics** | **Origin** |
| --- | --- | --- |
| **CHA** | Mucoid, cystic fibrosis isolate | Toussaint at al 1993 |
| Δ***pscF*** | CHA deleted of the *pscF* gene | Pastor et al 2005 |
| Δ***pscF/pscF*** | Δ*pscF* complemented with pIApG*-pscF* | Monlezun et al 2015 |
| Δ***pscF/pscF* N28S** | Δ*pscF* complemented with pIApG*-pscF* N28S | This study |
| Δ***pscF/pscF* D45A** | Δ*pscF* complemented with pIApG*-pscF* D45A | This study |
| Δ***pscF/pscF* P47A** | Δ*pscF* complemented with pIApG*-pscF* P47A | This study |
| Δ***pscF/pscF* Q54A** | Δ*pscF* complemented with pIApG*-pscF* Q54A | This study |
| Δ***pscF/pscF* R75A** | Δ*pscF* complemented with pIApG*-pscF* R75A | This study |
| Δ***pscF/pscF* D76A** | Δ*pscF* complemented with pIApG*-pscF* D76A | Quinaud et at 2007 |
| Δ***pscF/pscF* P47A/Q54A** | Δ*pscF* complemented with pIApG*-pscF* P47A/Q54A | This study |
| Δ***pcrV*** | CHA deleted of the *pcrV* gene | Goure et al 2004 |
| Δ***pcrV/pcrV*** | Δ*pcrV* complemented with pIApG*-pcrV* | Gebus et al 2008 |
|  |  |  |
| **PLASMIDS** | **Characteristics** | **Origin** |
| **pET22b-*pscF*** | *Nde*I-*Xho*I PCR fragment *pscF* in pET22b | Quinaud et at 2007 |
| **pET22b-*pscF* D76A** | pET22b-*pscF* introduces mutation D76A | This study |
| **pET22b-*pscF* P47A/Q54A** | pET22b-*pscF* introduces mutation P47A/Q54A | This study |
|  |  |  |
| **PRIMERS** | **Sequences** | **Characteristics** |
| **PscF-N28S-F** | 5’-CGTTGACGTCCTTGCTCGCTGCGTTGGC | PscF mutagenesis of residue N28 |
| **PscF-N28S-F** | 5’-GCCAACGCAGCGAGCAAGGACGTCAACG |  |
| **PscF-D45A-F** | 5’-CCGCCGGGTTGGCGGCATTGTCGGTCCCCT | PscF mutagenesis of residue D45 |
| **PscF-D45A-R** | 5’-AGGGGACCGACAATGCCGCCAACCCGGCGG |  |
| **PscF-P47A-F** | 5’-GCTCGGCCAGCAGCGCGGCGTTGTCGGCATTG | PscF mutagenesis of residue P47 |
| **PscF-P47A-R** | 5’-CAATGCCGACAACGCCGCGCTGCTGGCCGAGC |  |
| **PscF-Q54A-F** | 5’-CTTGTTGATCTTGTGGGCCAGCTCGGCC | PscF mutagenesis of residue Q54 |
| **PscF-Q54A-R** | 5’-GGCCGAGCTGGCCCACAAGATCAACAAG |  |
| **PscF-R75A-R** | 5’-CCTTGCATCAGGTCGGCCAGCGCACGGGTC | PscF mutagenesis of residue R75 |
| **PscF-R75A-F** | 5’-GACCCGTGCGCTGGCCGACCTGATGCAAGG |  |
| **PscF-D76A-F** | 5’-CGTGCGCTGCGCCGACCTGATGCAAGC | PscF mutagenesis of residue D76A |
| **PscF-D76A-R** | 5’-GCCTTGCATCAGGGCGCGGACCGCACG |  |
| **PscF-P47A/Q54A-F** | 5’-CCGACCACTTGTTGATCTTGTGTGCCAGCTCGG  CCAGCAGCGCCGCGTTGTCGGCATTGTCGGTCCCC | PscF mutagenesis of residues P47 and Q54 |
| **PscF-P47A/Q54A-R** | 5’-GGGGACCGACAATGCCGACAACGCGGCGCTG  CTGGCCGAGCTGGCACACAAGATCAACAAGTGGTCGG |  |

(Gébus et al., 2008; Monlezun et al., 2015; Toussaint et al., 1993)
