# Supplementary material for: Structural and Functional Characterization of the Type Three Secretion System (T3SS) Needle of Pseudomonas aeruginosa
Source: Front Microbiol. 2019 Mar 29;10:573. doi: 10.3389/fmicb.2019.00573 (PMC6455054; doi:10.3389/fmicb.2019.00573)
Supplement: Supplementary file 6 [file Data_Sheet_1.docx]

**Supplementary Material**

**TABLE S1:** A subset of the structural validation scores for the homology models before and after Chimera minimization. Significant improvements in clash score (overall and van der Waals) are observed for all models (best = zero). Scores calculated by the PSVS webserver.

| Subunit model | Chimera  minimization | MolProbity  Clash score | Ramachandran favored+allowed | Avg. VdW clashes |
| --- | --- | --- | --- | --- |
| PrgI  (2LPZ:A) | From PDB | 1.61 | 100% | 0.05 |
| PrgI  (reverse aligned) | pre | 86.29 | 97.4% | 2.68 |
|  | **post** | **12.10** | **96.2%** | **0.28** |
| PscF  (aligned) | pre | 79.44 | 97.6% | 2.46 |
|  | **post** | **5.94** | **100%** | **0.18** |
| PscF  (reverse aligned) | pre | 116.56 | 94.1% | 3.61 |
|  | **post** | **7.42** | **98.8%** | **0.23** |

**TABLE S2: Summary of strains, plasmids and primers used in this study.** Underlined nucleotides indicate the mutated amino acid.

| **STRAINS** | **Characteristics** | **Origin** |
| --- | --- | --- |
| **CHA** | Mucoid, cystic fibrosis isolate | Toussaint at al 1993 |
| Δ***pscF*** | CHA deleted of the *pscF* gene | Pastor et al 2005 |
| Δ***pscF/pscF*** | Δ*pscF* complemented with pIApG*-pscF* | Monlezun et al 2015 |
| Δ***pscF/pscF* N28S** | Δ*pscF* complemented with pIApG*-pscF* N28S | This study |
| Δ***pscF/pscF* D45A** | Δ*pscF* complemented with pIApG*-pscF* D45A | This study |
| Δ***pscF/pscF* P47A** | Δ*pscF* complemented with pIApG*-pscF* P47A | This study |
| Δ***pscF/pscF* Q54A** | Δ*pscF* complemented with pIApG*-pscF* Q54A | This study |
| Δ***pscF/pscF* R75A** | Δ*pscF* complemented with pIApG*-pscF* R75A | This study |
| Δ***pscF/pscF* D76A** | Δ*pscF* complemented with pIApG*-pscF* D76A | Quinaud et at 2007 |
| Δ***pscF/pscF* P47A/Q54A** | Δ*pscF* complemented with pIApG*-pscF* P47A/Q54A | This study |
| Δ***pcrV*** | CHA deleted of the *pcrV* gene | Goure et al 2004 |
| Δ***pcrV/pcrV*** | Δ*pcrV* complemented with pIApG*-pcrV* | Gebus et al 2008 |
|  |  |  |
| **PLASMIDS** | **Characteristics** | **Origin** |
| **pET22b-*pscF*** | *Nde*I-*Xho*I PCR fragment *pscF* in pET22b | Quinaud et at 2007 |
| **pET22b-*pscF* D76A** | pET22b-*pscF* introduces mutation D76A | This study |
| **pET22b-*pscF* P47A/Q54A** | pET22b-*pscF* introduces mutation P47A/Q54A | This study |
|  |  |  |
| **PRIMERS** | **Sequences** | **Characteristics** |
| **PscF-N28S-F** | 5’-CGTTGACGTCCTTGCTCGCTGCGTTGGC | PscF mutagenesis of residue N28 |
| **PscF-N28S-F** | 5’-GCCAACGCAGCGAGCAAGGACGTCAACG |  |
| **PscF-D45A-F** | 5’-CCGCCGGGTTGGCGGCATTGTCGGTCCCCT | PscF mutagenesis of residue D45 |
| **PscF-D45A-R** | 5’-AGGGGACCGACAATGCCGCCAACCCGGCGG |  |
| **PscF-P47A-F** | 5’-GCTCGGCCAGCAGCGCGGCGTTGTCGGCATTG | PscF mutagenesis of residue P47 |
| **PscF-P47A-R** | 5’-CAATGCCGACAACGCCGCGCTGCTGGCCGAGC |  |
| **PscF-Q54A-F** | 5’-CTTGTTGATCTTGTGGGCCAGCTCGGCC | PscF mutagenesis of residue Q54 |
| **PscF-Q54A-R** | 5’-GGCCGAGCTGGCCCACAAGATCAACAAG |  |
| **PscF-R75A-R** | 5’-CCTTGCATCAGGTCGGCCAGCGCACGGGTC | PscF mutagenesis of residue R75 |
| **PscF-R75A-F** | 5’-GACCCGTGCGCTGGCCGACCTGATGCAAGG |  |
| **PscF-D76A-F** | 5’-CGTGCGCTGCGCCGACCTGATGCAAGC | PscF mutagenesis of residue D76A |
| **PscF-D76A-R** | 5’-GCCTTGCATCAGGGCGCGGACCGCACG |  |
| **PscF-P47A/Q54A-F** | 5’-CCGACCACTTGTTGATCTTGTGTGCCAGCTCGG  CCAGCAGCGCCGCGTTGTCGGCATTGTCGGTCCCC | PscF mutagenesis of residues P47 and Q54 |
| **PscF-P47A/Q54A-R** | 5’-GGGGACCGACAATGCCGACAACGCGGCGCTG  CTGGCCGAGCTGGCACACAAGATCAACAAGTGGTCGG |  |

(Gébus et al., 2008; Monlezun et al., 2015; Toussaint et al., 1993)


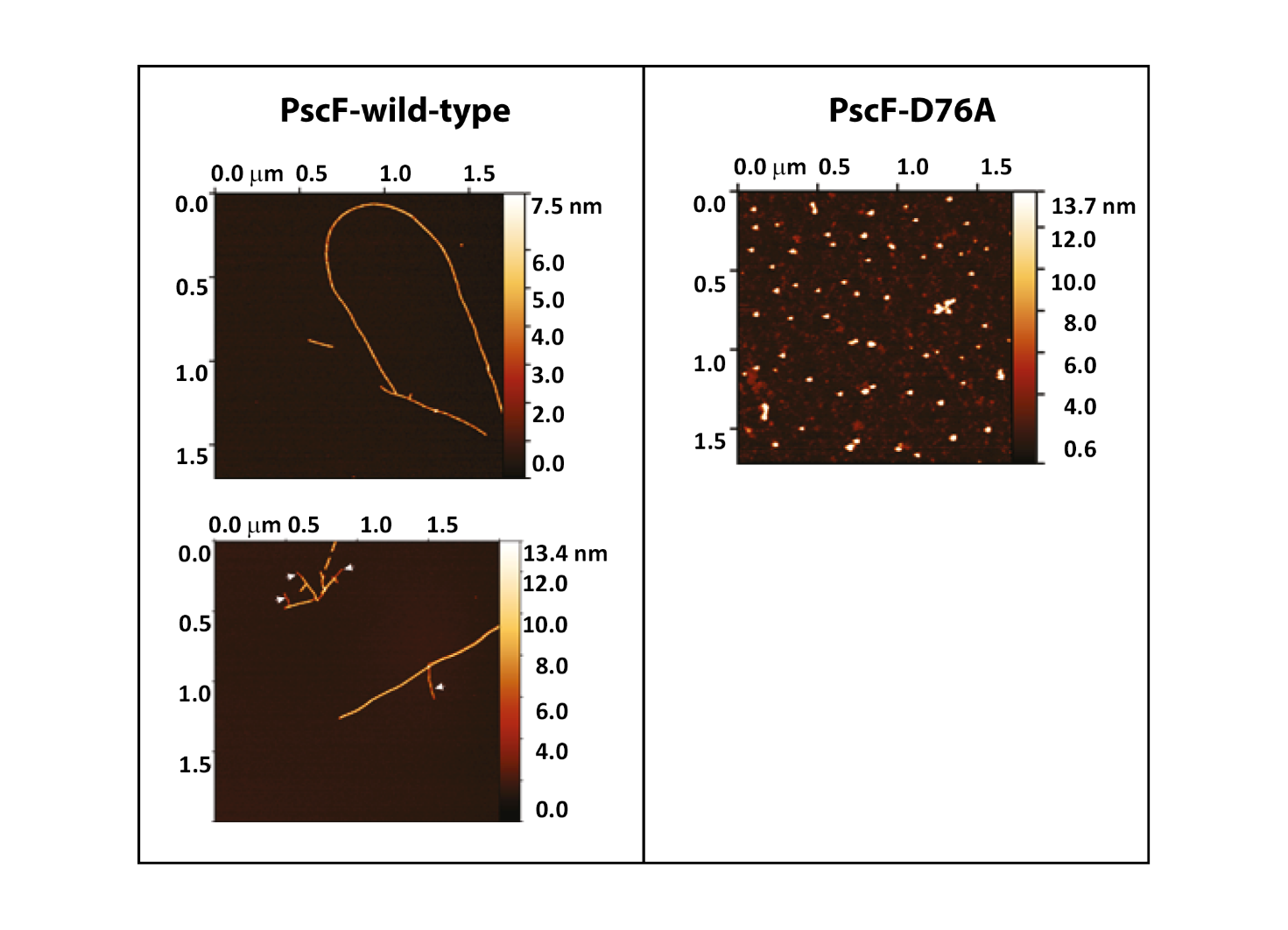


# FIGURE S1: AFM of purified PscF wild-type and PscF-D76A mutants. Height image of a PscF wild-type (left) and D76A mutant (right) using AFM on a 1.7 µm (top) or 2 µm (bottom) scan size. For wild-type PscF, sometimes single filaments with diameters of approx. 4.9 nm can be observed (dark orange indicated with white arrows). They seem to twist around each other to make a thicker assembly of about 8 nm in height. For the D76A mutant, no complete filament could be observed. The color bar represents the height of the image. Stripe noise has been removed using DeStripe and a median filter (3 px) has been applied for contrast improvement.

**
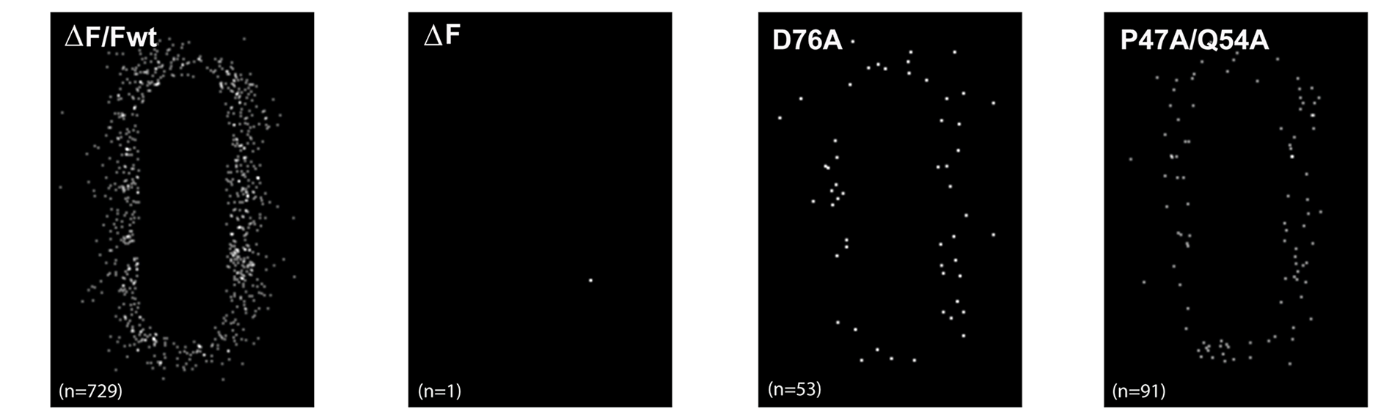
**

**FIGURE S2: Reconstitution of PcrV distribution around the *P. aeruginosa*** **ΔPscF, PscF-wild-type, -D76A and -P47A/Q54A variants.** Immunolabelled images with anti-PcrV shows in Fig 4. were analyzed with MicrobeJ. Projection of the PcrV foci onto a single cell for the different *P. aeruginosa* strains, using a threshold of 0.3 µm, n= number of spots in the image. *P. aeruginosa* ΔF and ΔF/Fwt were used as negative and positive control, respectively. Only few PcrV were visible on *P. aeruginosa* strains carrying a PscF-D76A or a PscF-P47A/Q54A compared to the PscF-wild-type strain.


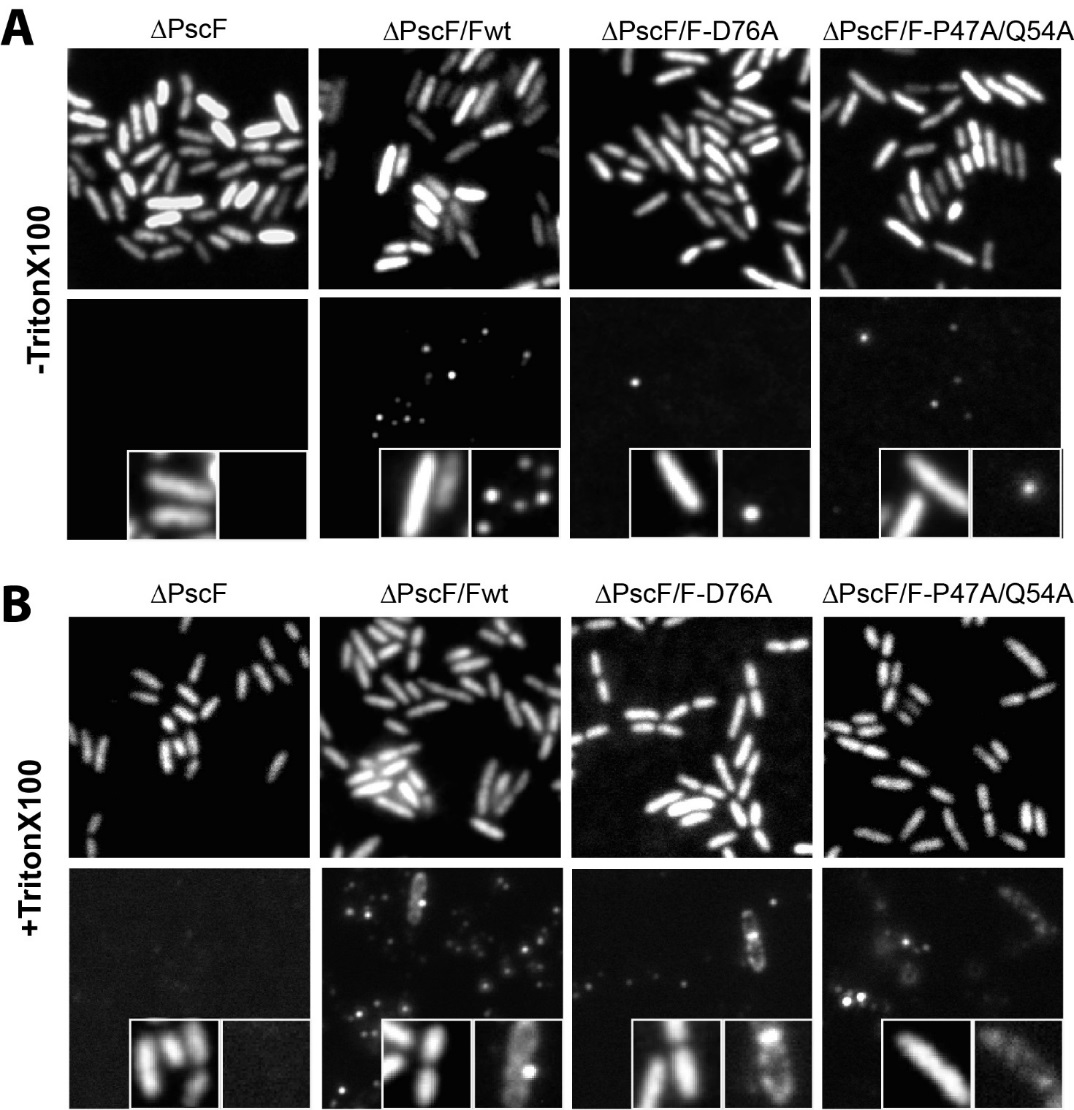


**FIGURE S3: Both *P. aeruginosa* PscF-D76A and P47A/Q54A strains express PscF.** *P. aeruginosa* cultures grown in T3SS-inducing conditions were fixed with 4% PFA and then visualized by florescence microscopy. The presence of PscF inside the D76A and P47A/Q54A strains was verified by permeabilizing the bacteria with 0.25 % Triton X-100 prior to PscF-antibody incubation. A) Without permeabilization treatment. B) After permeabilization. In each panel (top line) the labeling of bacteria by SYTO24-Green showing the bacterial integrity and; (bottom line) the labeling of PscF with anti-rabbit-Cy3 is reported. A magnification of the same zone with the two different labeling strategies are shown for each condition.
